# Once daily administration of the SGLT2 inhibitor, empagliflozin, attenuates markers of renal fibrosis without improving albuminuria in diabetic *db/db* mice

**DOI:** 10.1038/srep26428

**Published:** 2016-05-26

**Authors:** Linda A. Gallo, Micheal S. Ward, Amelia K. Fotheringham, Aowen Zhuang, Danielle J. Borg, Nicole B. Flemming, Ben M. Harvie, Toni L. Kinneally, Shang-Ming Yeh, Domenica A. McCarthy, Hermann Koepsell, Volker Vallon, Carol Pollock, Usha Panchapakesan, Josephine M. Forbes

**Affiliations:** 1 Glycation and Diabetes, Translational Research Institute, Mater Research Institute-University of Queensland, Woolloongabba, Queensland, Australia; 2 University of Queensland Biological Resources, St Lucia, Queensland, Australia; 3 School of Medicine, University of Queensland, St Lucia, Queensland, Australia; 4 Science and Engineering Faculty, Queensland University of Technology, Brisbane, Queensland, Australia; 5 Department of Molecular Plant Physiology and Biophysics, Julius-von-Sachs-Institute, University of Würzburg, Würzburg, Bavaria, Germany; 6 Departments of Medicine and Pharmacology, University of California San Diego, La Jolla, California, USA; 7 VA San Diego Healthcare System, San Diego, California, USA; 8 Department of Medicine, Kolling Institute of Medical Research, University of Sydney, St Leonards, New South Wales, Australia; 9 Mater Clinical School of Medicine, University of Queensland, South Brisbane, Queensland, Australia

## Abstract

Blood glucose control is the primary strategy to prevent complications in diabetes. At the onset of kidney disease, therapies that inhibit components of the renin angiotensin system (RAS) are also indicated, but these approaches are not wholly effective. Here, we show that once daily administration of the novel glucose lowering agent, empagliflozin, an SGLT2 inhibitor which targets the kidney to block glucose reabsorption, has the potential to improve kidney disease in type 2 diabetes. In male *db*/*db* mice, a 10-week treatment with empagliflozin attenuated the diabetes-induced upregulation of profibrotic gene markers, fibronectin and transforming-growth-factor-beta. Other molecular (collagen IV and connective tissue growth factor) and histological (tubulointerstitial total collagen and glomerular collagen IV accumulation) benefits were seen upon dual therapy with metformin. Albuminuria, urinary markers of tubule damage (kidney injury molecule-1, KIM-1 and neutrophil gelatinase-associated lipocalin, NGAL), kidney growth, and glomerulosclerosis, however, were not improved with empagliflozin or metformin, and plasma and intra-renal renin activity was enhanced with empagliflozin. In this model, blood glucose lowering with empagliflozin attenuated some molecular and histological markers of fibrosis but, as per treatment with metformin, did not provide complete renoprotection. Further research to refine the treatment regimen in type 2 diabetes and nephropathy is warranted.

Diabetic nephropathy accounts for 35–40% of new cases of end-stage renal disease in the developed world^1^,^2^. A major risk factor for the vascular complications of diabetes is chronic elevations in blood glucose concentrations (hyperglycemia) but there is no guarantee that glycemic control will prevent the onset and progression of micro- and/or macrovascular diseases^3^,^4^,^5^,^6^. At the first clinical sign of renal impairment (albuminuria), inhibitors of the renin-angiotensin system (RAS) are administered but they only slow progression of the disease^4^. Therefore, anti-diabetic strategies that effectively control blood glucose levels and prevent the onset and progression of diabetic nephropathy are in great demand.

Sodium-dependent glucose transporter (SGLT)-2 inhibitors, a new anti-diabetic strategy, target the renal proximal tubules to block glucose reabsorption, thereby enhancing urinary glucose excretion and conferring anti-hyperglycemic effects. They are indicated for use in individuals with type 2 diabetes (provided kidney function is at least moderate) and are under clinical investigation as an add-on to exogenous insulin in type 1 diabetes. Clinical studies with SGLT2 inhibitors have reported reductions in fasting plasma glucose and glycated hemoglobin (HbA_1c_) levels (0.7–0.8%) compared to placebo and other glucose lowering strategies^7^,^8^,^9^,^10^,^11^, and a reduction in cardiovascular mortality in individuals with type 2 diabetes and high cardiovascular risk^12^.

Under normal conditions, glucose is almost completely reabsorbed from the urinary filtrate by secondary active co-transporters located on the apical membrane, SGLT2 and SGLT1, in the early and late proximal tubule, respectively^13^. SGLT2 is responsible for the majority (up to 97%) of glucose reabsorption, while SGLT1 reabsorbs the majority of remaining luminal glucose. At the basolateral side, GLUT2 is responsible for the majority of glucose transport from the cells into the interstitium and peritubular circulation. In diabetes, the maximal threshold for glucose reabsorption is increased^14^,^15^. This contributes to hyperglycemia and, potentially, diabetic nephropathy via proximal tubular glucotoxicity.

While there is much focus on the role of glomeruli, tubulointerstitial changes more closely correlate with the clinical progression of nephropathy in diabetes^16^,^17^,^18^. Previous studies using human proximal tubular cells (HK2) reported that SGLT2 inhibition decreased the production of inflammatory and fibrotic markers induced by high glucose^19^. These *in vitro* findings suggest that SGLT2 inhibitors may provide renoprotection in diabetes by averting glucose from entering proximal tubule cells^20^,^21^. However, in recent preclinical studies, renoprotection with SGLT2 inhibition has been seen only when blood glucose levels were markedly improved^20^,^21^,^22^,^23^,^24^,^25^. Thus, the effect of SGLT2 inhibition on early kidney growth, inflammation, and fibrosis was proposed to result from blood glucose lowering^21^. The effect of SGLT2 inhibition on diabetic nephropathy, independent of blood glucose lowering, was assessed in diabetic eNOS knockout mice^26^. Blood glucose levels were matched between diabetic groups using insulin (group means >20 mmol/L) and, unlike an angiotensin receptor blocker, empagliflozin did not provide renoprotection. These data highlight that, in models of early diabetic nephropathy, renoprotection from hyperglycemia may be afforded only when circulating glucose levels and/or the activity of the RAS are sufficiently decreased.

In this study, we aimed to determine whether the administration of an SGLT2 inhibitor, empagliflozin, improves early manifestations of diabetic nephropathy in the *db/db* mouse model of type 2 diabetes. This model harbors a spontaneous mutation of the leptin receptor and is characterized by polyphagia, obesity, insulin resistance, hyperglycemia, pancreatic β-cell failure, and kidney and cardiovascular complications that are akin to type 2 diabetes in humans. We further aimed to determine whether the renoprotection offered by empagliflozin was associated with lowering of blood glucose concentrations, intrarenal RAS activity, and/or glucose content within kidney cortices. Whether these renal benefits were superior to the first-line, glucose-lowering therapy for type 2 diabetes, metformin, and/or additive upon empagliflozin and metformin dual therapy, were also assessed.

## Results

### Body weight and metabolic parameters

**Figure 1:**
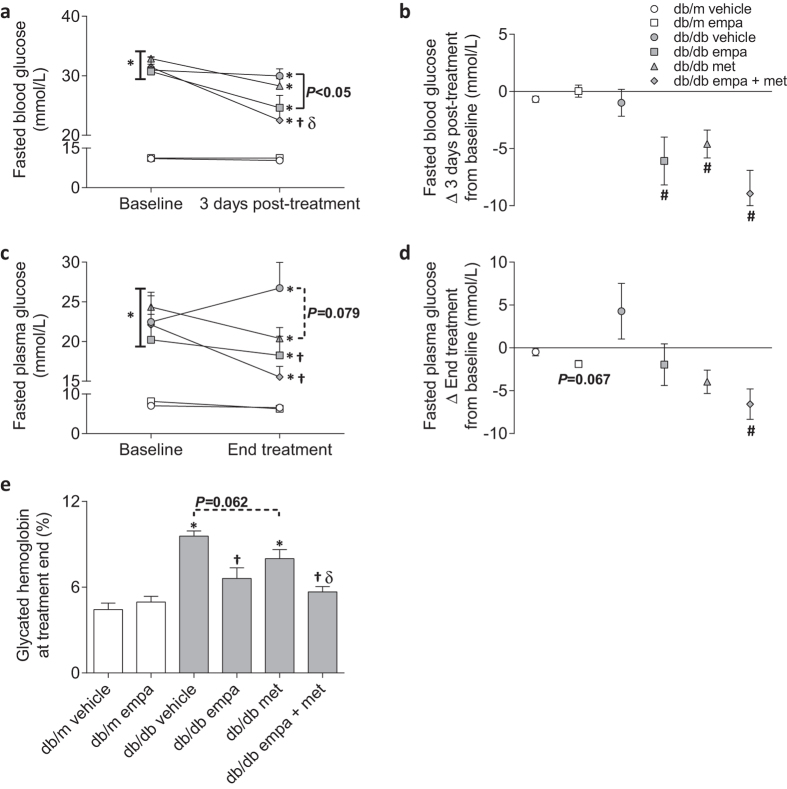
Circulating glucose levels. (**a,b**) Fasted blood glucose at baseline and three days after treatment start, (**c,d**) fasted plasma glucose at baseline and treatment end, and (**e**) glycated hemoglobin at treatment end in *db/m* (open) and *db/db* (grey) mice. Circles (ο) vehicle-treated; squares (◽) empagliflozin-treated; triangles (▵) metformin-treated; and diamonds (◊) empagliflozin + metformin co-treated. Data are means ± SEM (*n* = 5–11). (**a**,**c**,**e**): *P < 0.05 *vs db/m* vehicle, ^†^*P* < 0.05 *vs db/db* vehicle, ^δ^*P* < 0.05 *vs db/db* metformin within a time point by one-way ANOVA and Tukey’s post hoc. Comparisons by Student’s unpaired *t*-test: significance denoted by solid lines and trends denoted by dashed lines. (**b,d**): ^#^*P* < 0.05 delta change (▵) from baseline by Student paired *t*-test. *N.B.* (**a**,**b**): Some mice exceeded the upper limit of the glucometer, in which case the recorded blood glucose value was 33.3 mmol/L. Differences from baseline may therefore be underestimated.

**Figure 2:**
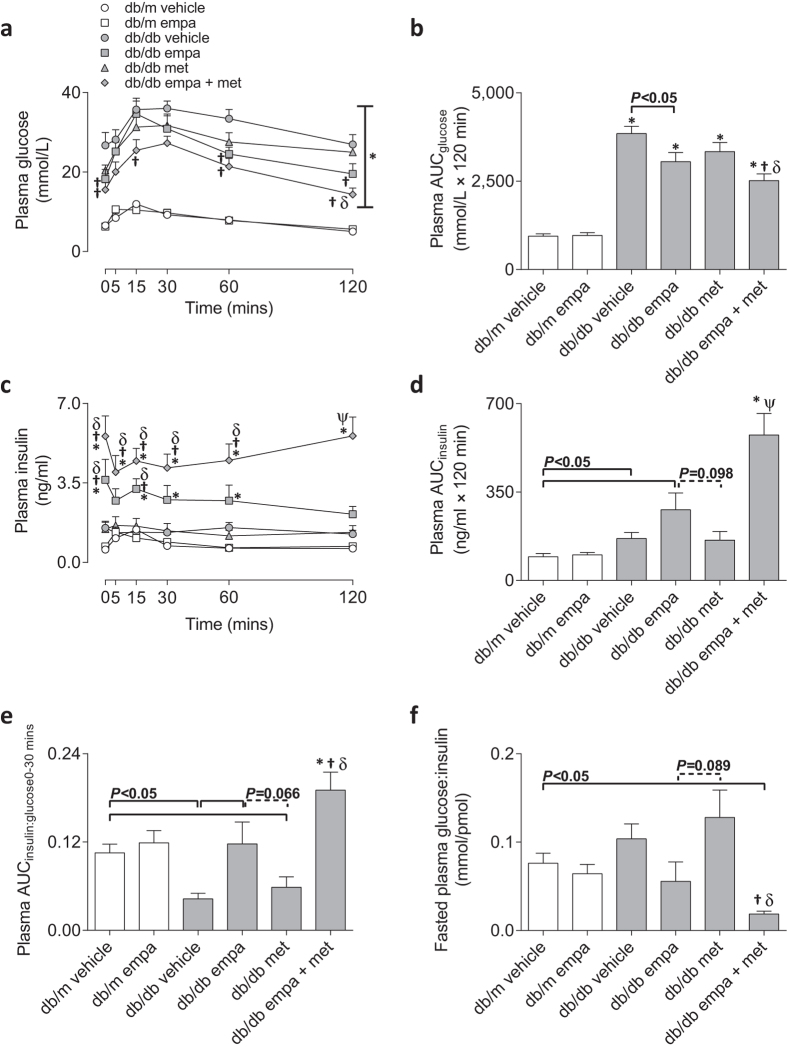
Oral glucose tolerance. (**a**) Plasma glucose concentrations over time, (**b**) area under glucose curve; AUCglucose, (**c**) plasma insulin concentrations over time, (**d**) area under insulin curve; AUCinsulin, and (**e**) insulinogenic index; AUCinsulin:glucose 0–30 mins in response to an oral glucose bolus (2 g/kg body weight) and (**f**) fasted plasma glucose-to-insulin ratio (*t* = 0 mins) in *db/m* (open) and *db/db* (grey) mice. Circles (ο) vehicle-treated; squares (◽) empagliflozin-treated; triangles (▵) metformin-treated; and diamonds (◊) empagliflozin + metformin co-treated. Data are means ± SEM (*n* = 6–11). *P < 0.05 *vs db/m* vehicle, ^†^*P* < 0.05 *vs db/db* vehicle, ^δ^*P* < 0.05 *vs db/db* metformin, ^Ψ^*P* < 0.05 *vs* all other *db/db* groups by one-way ANOVA and Tukey’s post hoc. Comparisons by Student’s unpaired *t*-test: significance denoted by solid lines and trends denoted by dashed lines.

In this study, *db/db* and *db/m* littermates were treated with empagliflozin (10 mg/kg/day) or vehicle by single daily oral gavage for 10 weeks. Two additional *db/db* groups were included and treated with the first-line anti-diabetic agent, metformin (250 mg/kg/day), or empagliflozin and metformin co-therapy (as per mono-therapy dosages). At treatment commencement (baseline; 10 weeks of age), *db/db* mice were ~14 g heavier (>1.5-fold, Table 1) with higher fasting blood and plasma glucose concentrations compared to non-diabetic *db/m* mice (Fig. 1a,c). Between weeks five and 10 of treatment, *db/db* mice administered with vehicle or metformin lost 3.0 and 4.4 g, respectively, whilst empagliflozin monotherapy maintained body weight and co-treated mice gained 3.1 g (Table 1). This may relate to differences in circulating insulin levels (described below in *Oral glucose tolerance test (OGTT)*; Fig. 2). For *db/m* mice, both vehicle- (*P* = 0.07) and empagliflozin-treated groups gained 1.0 g between weeks five and 10 of treatment (Table 1). At 10 weeks of treatment (20 weeks of age), *db/db* mice treated with empagliflozin were 8 to 11 g heavier than vehicle- and metformin-only-treated mice (Table 1).

Blood and plasma glucose concentrations were measured 20–24 h after the previous day’s therapy. Three days after the commencement of treatment, all mice treated with empagliflozin had lower fasting blood glucose levels compared to *db/db* vehicle and all intervention arms had considerable reductions from baseline (Fig. 1a,b). By the study end, SGLT2 inhibitor monotherapy lowered fasting plasma glucose levels to 18 mmol/L compared to 27 mmol/L in *db/db* vehicle (Fig. 1c). In addition, glycated hemoglobin was restored to non-diabetic levels, achieving 6.6% compared to 9.6% in *db/db* vehicle (Fig. 1e). Co-therapy with metformin provided modest incremental benefits, lowering fasting plasma glucose to 16 mmol/L, which was also reduced from baseline, and glycated hemoglobin to 5.7% (Fig. 1c–e). Metformin monotherapy tended to reduce fasting plasma glucose (20 mmol/L) and glycated hemoglobin (8.0%) compared to *db/db* vehicle (*P* = 0.079 and *P* = 0.062, respectively, Fig. 1c,e).

All *db/db* mice consumed more food and water, and produced more urine compared to *db/m* when assessed at two and six weeks into the treatment period (Table 1). There were no effects of treatment on food intake but, in the *db/m* mice, empagliflozin increased water consumption at six weeks (*P* = 0.066) and urine output at both ages (two weeks *P* = 0.055, Table 1).

### Oral glucose tolerance test (OGTT)

An OGTT was performed on all mice 20–24 h after the previous day’s treatment. Compared to *db/m*, all *db/db* mice had elevated plasma glucose concentrations for the duration of the OGTT resulting in a higher area under the glucose curve (AUC_glucose_, Fig. 2a,b). Empagliflozin monotherapy reduced plasma glucose levels at baseline and at 60 and 120 mins after the glucose bolus compared to vehicle *db/db* mice, resulting in a reduced AUC_glucose_ (Fig. 2a,b). Co-therapy decreased plasma glucose concentrations at baseline, and at 15, 60, and 120 mins of the OGTT, and reduced AUC_glucose_ compared to vehicle- and metformin-treated *db/db* mice (Fig. 2a,b). Metformin monotherapy did not improved glucose tolerance during the OGTT (Fig. 2a,b).

Plasma insulin concentrations were increased throughout the OGTT in co-treated mice and at specific time points (0, 15, 30, and 60 mins) in empagliflozin mono-treated mice compared to *db/m* (Fig. 2c). Co-therapy also resulted in elevated plasma insulin levels throughout the OGTT when compared to diabetic vehicle- and metformin-treated mice, whilst empagliflozin increased insulin at baseline and 15 mins only (Fig. 2c). Vehicle-, empagliflozin-, and co-treated diabetic mice had elevated area under the insulin curve (AUC_insulin_) compared to non-diabetic and this difference was most pronounced for the latter, which also had enhanced insulin response when compared to all other *db/db* groups (Fig. 2d). Vehicle- and metformin-treated *db/db* mice exhibited a 50–60% reduction in the insulinogenic index (AUC_insulin:glucose 0–30 mins_), and empagliflozin, as a single and dual therapy with metformin, restored and further increased this index compared to *db/m* levels, respectively (Fig. 2e). Fasting plasma glucose-to-insulin ratio was reduced in the co-treated mice when compared to vehicle- and metformin-treated diabetic mice as well as non-diabetic *db/m* mice (Fig. 2f), indicative of reduced insulin sensitivity. Empagliflozin mono-therapy also tended to reduce this ratio when compared to the metformin-treated diabetic arm (*P* = 0.089, Fig. 2f). HOMA-IR was elevated in all diabetic mice and exacerbated in the co-treated group (see Supplementary Fig. S1a). Insulin positivity within pancreatic islets was not different between non-diabetic and diabetic vehicle-treated mice (see Supplementary Fig. S1b). Co-therapy, however, increased insulin positive staining in *db/db* mice compared to *db/m* vehicle and other diabetic groups, whilst metformin reduced insulin positivity compared to vehicle-treated counterparts (see Supplementary Fig. S1b). Empagliflozin increased islet insulin content when administered to *db/m* mice (see Supplementary Fig. S1b).

### Renal glucose handling, and expression of glucose transporters and gluconeogenic enzymes

**Figure 3:**
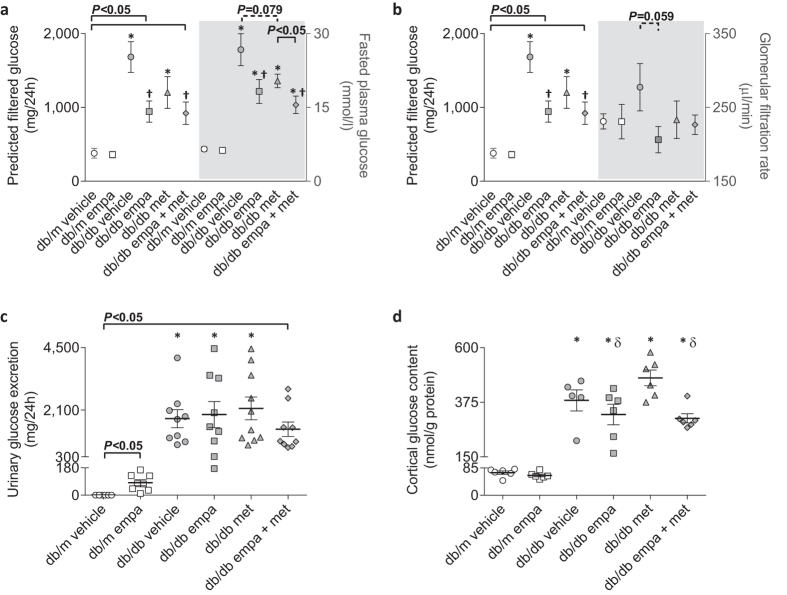
Filtered glucose load, glucosuria, and cortical glucose content. Predicted filtered glucose load determined by (**a**) fasted plasma glucose and (**b**) GFR on dual y-axes figures, (**c**) urinary glucose excretion, and (**d**) cytosolic glucose levels within kidney cortices in *db/m* (open) and *db/db* (grey) mice. Circles (ο) vehicle-treated; squares (◽) empagliflozin-treated; triangles (▵) metformin-treated; and diamonds (◊) empagliflozin + metformin co-treated. Data are (**a,b**) means ± SEM or (**c,d**) individual mice with means ± SEM (*n* = 5–11). *P < 0.05 *vs db/m* vehicle, ^†^*P* < 0.05 *vs db/db* vehicle, ^δ^*P* < 0.05 *vs db/db* metformin by one-way ANOVA and Tukey’s post hoc. Comparisons by Student’s unpaired *t*-test: significance denoted by solid lines and trends denoted by dashed lines.

The predicted filtered glucose load, assessed after eight weeks of treatment, was increased in all *db/db* mice (Fig. 3a,b). Empagliflozin (mono- and co-therapy) reduced the filtered glucose load by 45% compared to *db/db* vehicle, owing to a similar reduction in fasted plasma glucose levels (Fig. 3a). In *db/db* mice treated with empagliflozin monotherapy, reduced filtered glucose load was also mediated by a modest reduction in glomerular filtration rate (GFR; *P* = 0.059, Fig. 3b). All *db/db* mice had glucosuria, excreting ~1500 mg glucose into their urine each day, compared with <0.2 mg in *db/m* vehicle (Fig. 3c). Empagliflozin in non-diabetic mice increased urinary glucose excretion to >80 mg per day (Fig. 3c) without affecting circulating glucose concentrations compared to vehicle counterparts (see above; Fig. 1). In mice with diabetes, empagliflozin monotherapy caused a left-ward shift in the relationship between plasma glucose level and urinary glucose, indicating that urinary glucose excretion was greater for any given concentration of plasma glucose (see Supplementary Fig. S2a). This treatment effect was lost when empagliflozin was co-administered with metformin and absent in metformin-treated mice (see Supplementary Fig. S2b,c). These observations with respect to glucosuria, measured after six weeks of treatment, were also evident after two weeks of treatment (data not shown). Cytosolic glucose concentrations within renal cortices, determined in tissue that was harvested from fasted mice ~24 h after the last dose, were increased in all *db/db* mice compared to *db/m*, and exacerbated by metformin mono-therapy (Fig. 3d). Empagliflozin, either as a mono-therapy or co-therapy with metformin, did not reduce cortical glucose content (Fig. 3d).

**Figure 4:**
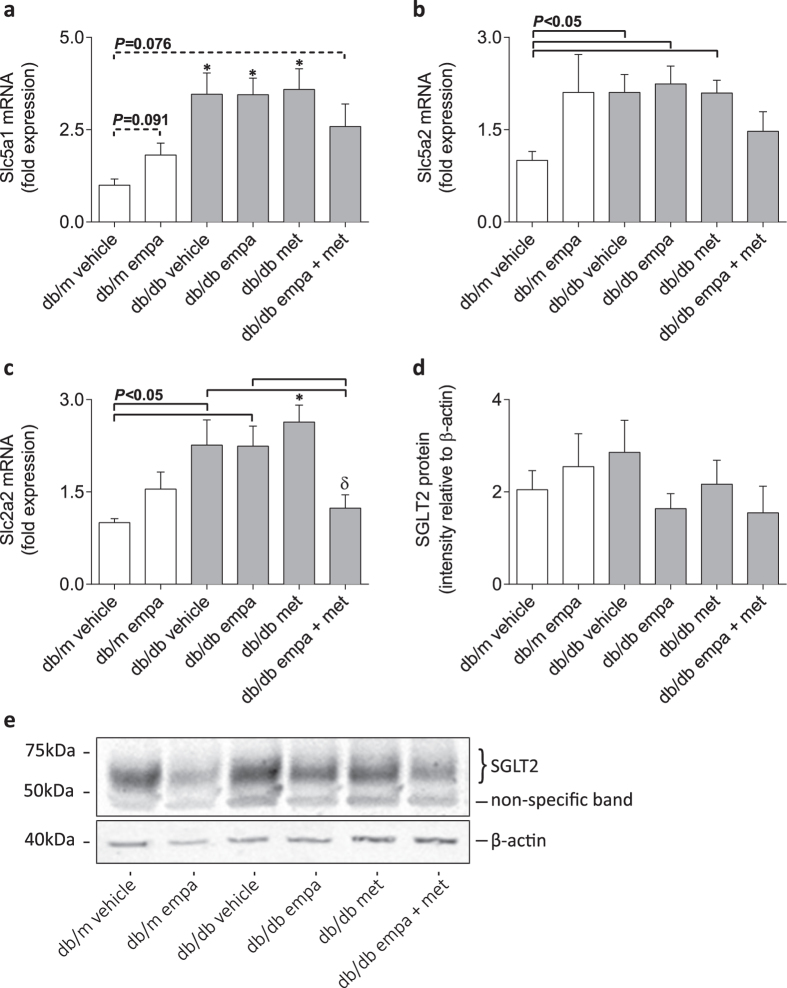
mRNA and protein expression of glucose transporters in kidney cortices. (**a–c**) Real-time qPCR for *Slc5a1* (encoding SGLT1), *Slc5a2* (encoding SGLT2), and *Slc2a2* (encoding GLUT2), and (**d,e**) Western immunoblot for total cell membrane SGLT2 expression in *db/m* (open) and *db/db* (grey) mice. Data are means ± SEM (*n* = 4–6). *P < 0.05 *vs db/m* vehicle, ^δ^*P* < 0.05 *vs db/db* metformin by one-way ANOVA and Tukey’s post hoc. Comparisons by Student’s unpaired *t*-test: significance denoted by solid lines and trends denoted by dashed lines. Blot has been cropped to improve clarity; see Supplementary Fig. S3 for full length blots.

Compared with *db/m* mice, the expression of genes encoding SGLT1 (*Slc5a1*), SGLT2 (*Slc5a2*), and GLUT2 (*Slc2a2*) were elevated in all diabetic mice, except for those administered co-therapy (Fig. 4a–c). Co-treatment substantially reduced the diabetes-induced upregulation of *Slc2a2*, but not *Slc5a1* and *Slc5a2*, mRNA levels when compared to all other diabetic arms (Fig. 4a–c). In non-diabetic *db/m* mice, empagliflozin tended to increase gene expression of *Slc5a1* (*P* = 0.091), but did not affect *Slc5a2* and *Slc2a2* expression (Fig. 4a–c). Renal cortical protein concentration of SGLT2, in total cell membranes, was not different between groups (Fig. 4d,e).

**Figure 5:**
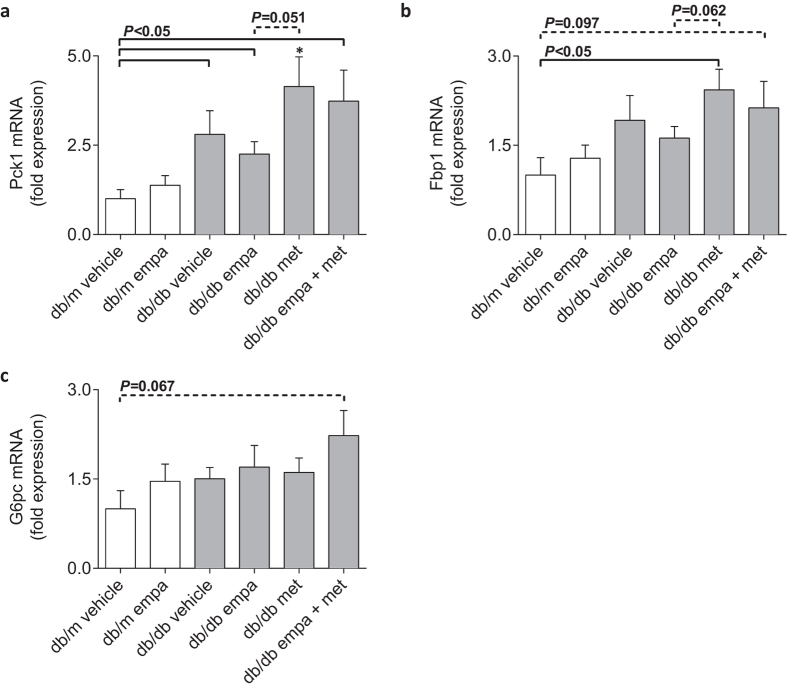
mRNA expression of gluconeogenic enzymes in kidney cortices. (**a–c**) Real-time qPCR for *Pck1* (encoding phosphoenolpyruvate carboxykinase 1; PEPCK), *Fbp1* (encoding fructose bisphosphatase 1), and *G6pc* (encoding glucose-6-phosphatase) in *db/m* (open) and *db/db* (grey) mice. Data are means ± SEM (*n* = 4–6). *P < 0.05 *vs db/m* vehicle by one-way ANOVA and Tukey’s post hoc. Comparisons by Student’s unpaired *t*-test: significance denoted by solid lines and trends denoted by dashed lines.

Renal cortical expression of *Pck1*, encoding a key gluconeogenic enzyme, PEPCK, was increased in all *db/db* groups (Fig. 5a). *Fbp1*, which encodes the rate-limiting fructose-1,6-bisphosphatase 1 enzyme, was also increased in metformin and tended to be increased in co-treated (*P* = 0.097) diabetic mice (Fig. 5b). *G6pc*, encoding for the enzyme, glucose-6-phophatase, tended to be increased in co-treated mice only (*P* = 0.067, Fig. 5c). Metformin-treated *db/db* mice tended to have greater expression of *Pck1* and *Fbp1* when compared to diabetic mice treated with empagliflozin (*P* = 0.051 and *P* = 0.062, Fig. 5a,b).

### Renal function

**Figure 6:**
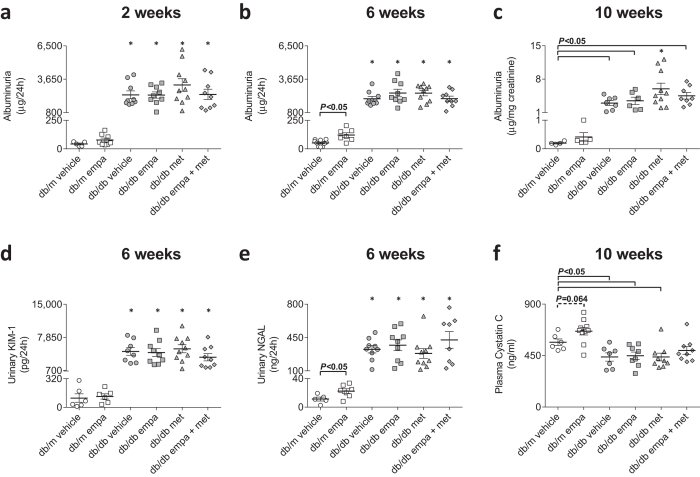
Kidney function. (**a–c**) Albuminuria, (**d**) urinary concentrations of kidney injury molecule 1 (KIM-1), (**e**) neutrophil gelatinase-associated lipocalin (NGAL), and (**f**) plasma cystatin C levels in *db/m* (open) and *db/db* (grey) mice. Circles (ο) vehicle-treated; squares (◽) empagliflozin-treated; triangles (▵) metformin-treated; and diamonds (◊) empagliflozin + metformin co-treated. Weeks refer to treatment duration. Data are individual mice with means ± SEM (*n* = 5–10). *P < 0.05 *vs db/m* vehicle, ^δ^*P* < 0.05 *vs db/db* metformin by one-way ANOVA and Tukey’s post hoc. Comparisons by Student’s unpaired *t*-test: significance denoted by solid lines and trends denoted by dashed lines. Headings refer to duration of treatment.

All *db/db* mice had albuminuria at two, six and 10 weeks of treatment which remained unaffected by any treatment regimen (Fig. 6a–c). Empagliflozin in the non-diabetic *db/m* mice increased urinary albumin excretion by >2-fold at 6 weeks of treatment (Fig. 6b), due to a similar increase in urine production (see above; Table 1). Urinary excretion of renal tubule damage markers, KIM-1 and NGAL, were ~40 times higher in *db/db vs db/m*, without an effect of treatment (Fig. 6d,e). Urinary NGAL in non-diabetic mice treated with empagliflozin was also increased; attributed to increased urine production (see above; Table 1). In all *db/db* mice, except those treated with the combination therapy, plasma cystatin C was reduced by ~22%, indicative of glomerular hyperfiltration (Fig. 6f). In *db/m* mice, empagliflozin tended to increase plasma cystatin C levels (+ 17%, P = 0.064, Fig. 6f), suggesting a small decrease in GFR.

### Renal morphology and expression of profibrotic genes

**Figure 7:**
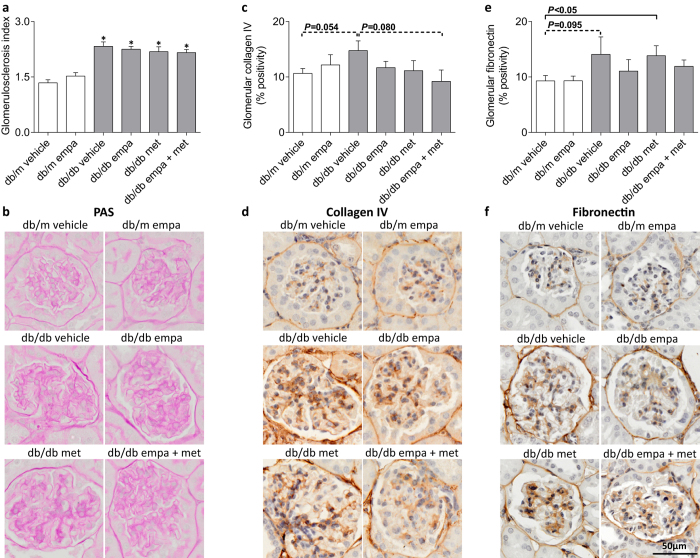
Glomerulosclerosis and mesangial expansion. (**a,b**) Periodic acid-Schiff (PAS) stain for glomerulosclerosis, and (**c,d**) collagen IV and (**e,f**) fibronectin immunostaining in glomeruli in *db/m* (open) and *db/db* (grey) mice. Data are means ± SEM (*n* = 5–9). *P < 0.05 *vs db/m* vehicle by one-way ANOVA and Tukey’s post hoc. Comparisons by Student’s unpaired *t*-test: significance denoted by solid lines and trends denoted by dashed lines.

**Figure 8:**
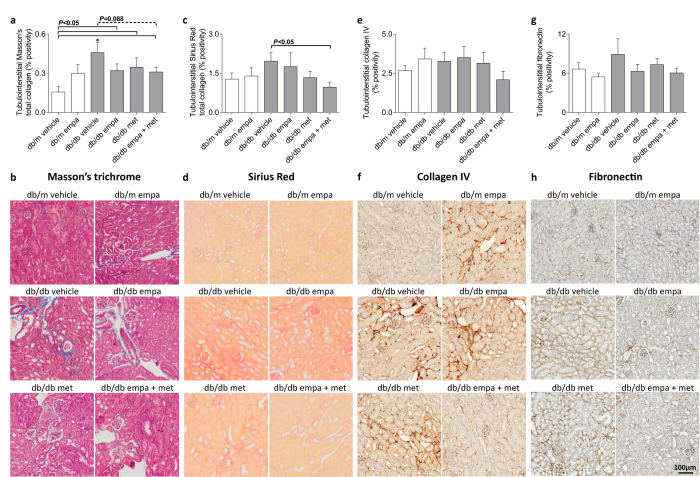
Tubulointerstitial fibrosis. (**a,b**) Masson’s trichrome, (**c,d**) Sirius Red, and (**e,f**) collagen IV and (**g,h**) fibronectin immunostaining in tubulointerstitium in *db/m* (open) and *db/db* (grey) mice. Data are means ± SEM (*n* = 5–10). *P < 0.05 *vs db/m* vehicle by one-way ANOVA and Tukey’s post hoc. Comparisons by Student’s unpaired *t*-test: significance denoted by solid lines and trends denoted by dashed lines.

Compared to *db/m*, kidney weight was ~23% greater in *db/db* vehicle-, empagliflozin-, and co-treated mice but 43% greater in metformin-treated mice (Table 1). Glomerulosclerosis (PAS-positive staining) was elevated in all *db/db* mice and unaffected by treatment (Fig. 7a,b). Glomerular collagen IV and fibronectin accumulation tended to be higher in vehicle-treated diabetic *vs* non-diabetic mice (*P* = 0.054 and *P* = 0.095, respectively, Fig. 7c–f). This diabetes-induced deposition of glomerular collagen IV and fibronectin was absent in all treated arms, except for the latter which remained elevated in metformin-treated mice (Fig. 7c-f). Total collagen accumulation within renal cortical/outer medullary regions was increased in all *db/db* mice compared to *db/m* when quantified using Masson’s trichrome (*P* < 0.05), but not Sirius Red, staining (Fig. 8a–d). Co-treatment with empagliflozin and metformin tended to reduce Masson’s trichrome positivity (*P* = 0.088) and significantly decreased Sirius Red staining when compared to *db/db* vehicle (Fig. 8a–d). The degree of tubulointerstitial collagen IV and fibronectin staining, however, was not different among groups (Fig. 8e–h).

**Figure 9:**
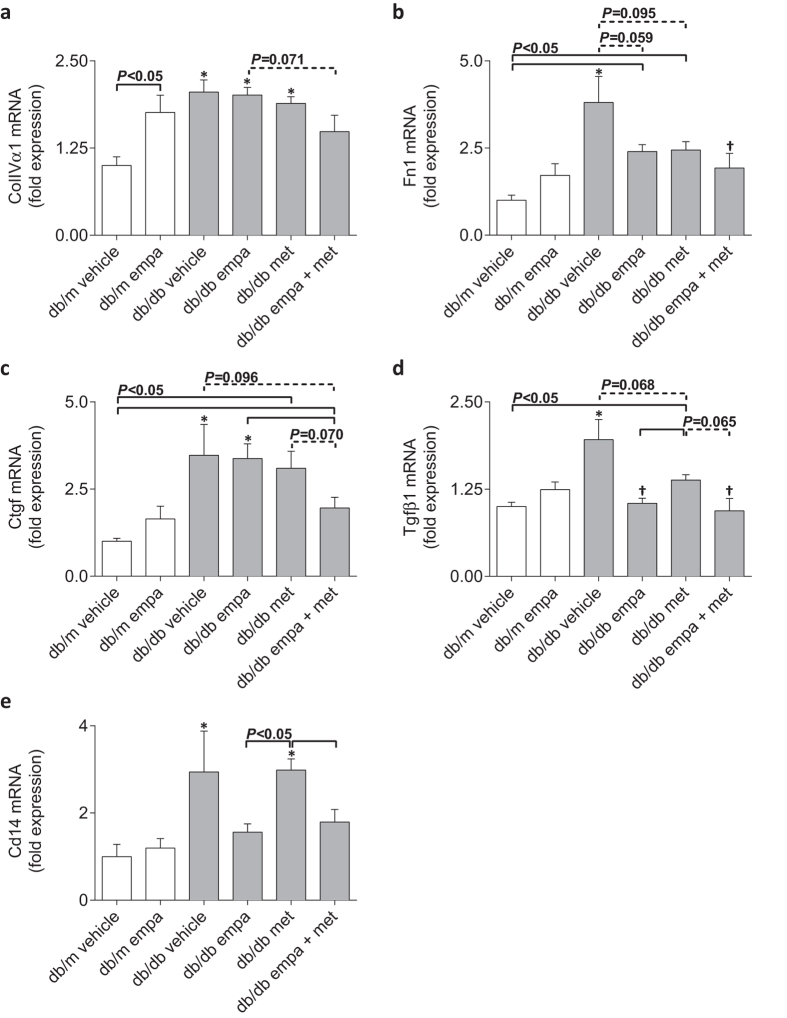
Renal cortical expression of profibrotic genes and macrophage marker. Real-time qPCR for (**a**) *ColIV*α*1* (encoding collagen type IVα1), (**b**) *Fn1* (encoding fibronectin), (**c**) *Ctgf* (encoding connective tissue growth factor, (**d**) *Tgf* β*1* (encoding transforming growth factor β1), and (**e**) *Cd14* (encoding CD14) in *db/m* (open) and *db/db* (grey) mice. Data are means ± SEM (*n* = 4–6). *P < 0.05 *vs db/m* vehicle, ^†^*P* < 0.05 *vs db/db* vehicle by one-way ANOVA and Tukey’s post hoc. Comparisons by Student’s unpaired *t*-test: significance denoted by solid lines and trends denoted by dashed lines.

Renal cortical expression of *ColIVα1, Fn1, Ctgf, Tgfβ1*, and the cell surface macrophage marker, *Cd14*, were elevated in vehicle-treated diabetic *vs* non-diabetic mice (Fig. 9a–e). In mice treated with empagliflozin, the diabetes-induced upregulation of *Tgfβ1* and *Cd14* was absent and there was a trend for reduced *Fn1* expression compared to vehicle-treated *db/db* mice (*P* = 0.059, Fig. 9b,d,e). *ColIVα* and *Ctgf*, however, remained elevated in empagliflozin-treated diabetic *vs* non-diabetic mice (Fig. 9a,c). Metformin did not restore the diabetes-induced upregulation of any genes but tended to decrease *Fn1* (*P* = 0.095) and *Tgfβ1* (*P* = 0.068) compared to *db/db* vehicle (Fig. 9a–e). Co-administration of empagliflozin and metformin provided the greatest benefits, such that the diabetes-induced upregulation of all genes was no longer present in this group, except for *Ctgf* expression which remained elevated compared to non-diabetic levels (Fig. 9a–e). *Ctgf* expression was, however, reduced when compared to other *db/db* groups (*P* = 0.096 *vs* vehicle, *P* < 0.05 *vs* empagliflozin, *P* = 0.070 *vs* metformin, Fig. 9c). Of note, in non-diabetic mice, empagliflozin increased the renal cortical expression of *ColIVα1* but did not affect the expression of any other genes (Fig. 9a–e).

### Plasma renin activity, and intra-renal renin activity and angiotensin II content

**Figure 10:**
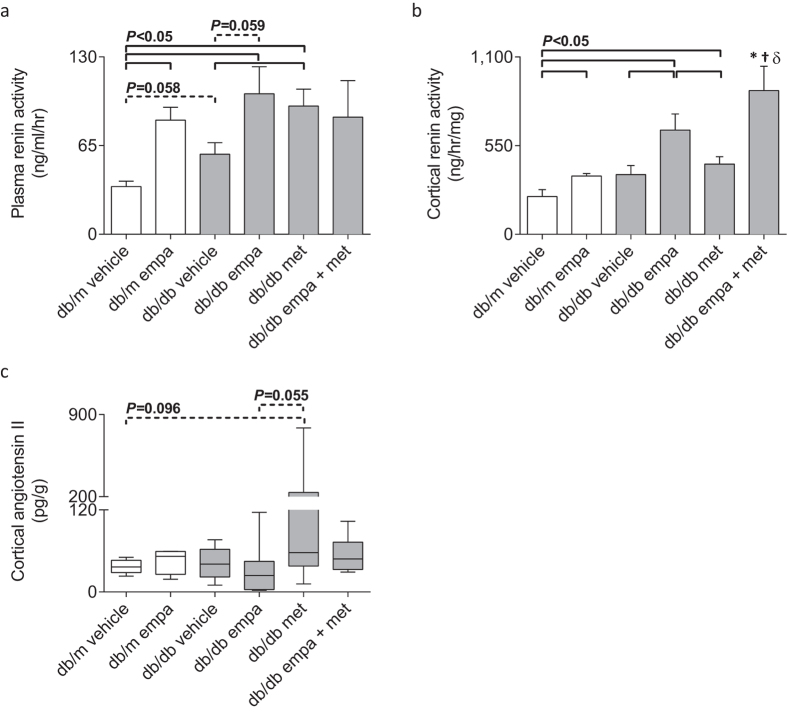
Circulating and intra-renal RAS. (**a**) Plasma renin activity, and renal cortical (**b**) renin activity and (**c**) angiotensin II content in *db/m* (open) and *db/db* (grey) mice. Data are (**a,b**) means ± SEM and (**c**) median ± IQR with min and max values (*n* = 5–10). *P < 0.05 *vs db/m* vehicle, ^†^*P* < 0.05 *vs db/db* vehicle, ^δ^*P* < 0.05 *vs db/db* metformin by one-way ANOVA and Tukey’s post hoc. (**c**) Analyzed by Kruskal-Wallis one-way ANOVA and no significant differences observed. Comparisons by Student’s unpaired *t*-test: significance denoted by solid lines and trends denoted by dashed lines.

Plasma renin activity tended to increase in diabetic *vs* non-diabetic vehicle-treated counterparts (*P* = 0.058, Fig. 10a). Empagliflozin treatment increased plasma renin activity in non-diabetic mice, and both empagliflozin (*P* = 0.059) and metformin mono-therapies exacerbated this diabetes-induced increase (Fig. 10a). Renin activity in renal cortices was not different between non-diabetic and diabetic mice administered with vehicle (Fig. 10b). However, empagliflozin increased intra-renal renin activity levels in both non-diabetic and diabetic mice (Fig. 10b). Combination therapy exacerbated this increase in cortical renin activity but metformin mono-therapy had no effect compared to vehicle-treated diabetic mice (Fig. 10b). Renal cortical levels of angiotensin II were not different between *db/m* and *db/db* vehicle-treated mice, however, an increasing trend was seen in the metformin-treated group *vs db/m* vehicle (*P* = 0.096) and *db/db* empagliflozin (*P* = 0.055, Fig. 10c).

## Discussion

In the present study, we demonstrate in the *db/db* mouse model of type 2 diabetes that upregulation of some profibrotic genes in the kidney was ameliorated upon SGLT2 inhibition, parallel to the effects of metformin. When empagliflozin and metformin were co-administered, additional molecular and histological markers of kidney fibrosis were attenuated. Diabetes-induced upregulation of renal *Cd14* was also no longer present in the mice treated with empagliflozin (mono- and co-treatment with metformin), but not metformin, suggesting that the aforementioned benefits with SGLT2 inhibition may have been mediated through reduced inflammation. However, empagliflozin did not improve diabetes-induced albuminuria, increased urinary markers of tubule damage (KIM-1 and NGAL), renal hypertrophy, or glomerulosclerosis, when administered alone or in combination with metformin. These partial benefits occurred in line with a modest lowering of blood glucose that remained above non-diabetic levels. Thus, in light of our findings and others^26^, who have failed to observe complete restoration of kidney function, the determinants of renoprotection with SGLT2 inhibition in diabetes warrant further consideration.

Previously, in *db/db* mice, the administration of an SGLT2 inhibitor; dapagliflozin in males^23^ and tofogliflozin in females^25^, prevented progressive albuminuria, parallel to the effect of losartan in the latter study, and lowered plasma glucose levels to <15 mmol/L. In male *db/db* mice treated with empagliflozin, Lin *et al.* similarly demonstrated reduced albuminuria and glomerulosclerosis, associated with complete amelioration of hyperglycemia^24^. Vallon *et al.*, also demonstrated that empagliflozin administered to male Akita/+ mice, a model of type 1 diabetes, reduced albuminuria, renal hypertrophy, and markers of inflammation, which was in proportion to blood glucose lowering (average ~11 mmol/L)^21^. The timing and degree of blood glucose lowering, and dose of therapy are the major differences between the current study and previous reports of significant renoprotection. We commenced treatment two to three weeks later than that of Terami *et al.*^23^, Lin *et al.*^24^, and Nagata *et al.*^25^, and plasma glucose remained >15 mmol/L in our study. The dose of empagliflozin administered to Akita/+ mice in Vallon *et al.* and *db/db* mice in Lin *et al.* equates to four to six times that of the current study, which was provided *ad libitum* in food, and likely contributes to the pronounced blood glucose lowering and renoprotection seen in those studies^21^,^24^. Despite restoration of glycated hemoglobin levels with empagliflozin in our study, it is likely that plasma glucose levels fluctuated around the single daily administration, reaching spikes of 18 and 16 mmol/L in mono- and co-treated groups, respectively, compared to 6.5 mmol/L in non-diabetic vehicle-treated mice. We determined the circulating glucose level 20–24 h after the previous day’s gavage; likely at a peak glucose concentration given that empagliflozin half-life is ~5.6 h in the male mouse. Indeed, the benefits of early and intensive blood glucose lowering for microvascular complications are well established^27^,^28^ and fluctuations in glucose level are known to increase the risk of complications independent of average glucose exposure^29^. It is therefore likely that renal structures remained exposed to hyperglycemia, at least temporally, in this study. We did not assess blood glucose variation by continuous monitoring in the present study. In type 2 diabetic patients, twice daily treatment with empagliflozin as an add-on to metformin was not superior to once daily treatment in terms of blood glucose lowering^30^. However, long-term microvascular outcomes from this treatment regimen are yet to be determined. Future pre-clinical and clinical studies on renal outcomes would benefit from early and multiple daily dosing of empagliflozin, continuous glucose monitoring, and the risk to benefit ratio of higher doses closely monitored.

We observed that SGLT2 inhibition in *db/db* mice reduced the filtered glucose load which, based on previous *in vitro* findings, was expected to downregulate markers of proximal tubular damage^19^,^31^,^32^,^33^, and translate into functional improvements for the kidney. However, clinical features of diabetic nephropathy, including albuminuria, urinary markers of tubule damage (KIM-1 and NGAL), kidney growth, and glomerulosclerosis were not improved in this study, even when empagliflozin was co-administered with metformin. Empagliflozin is expected to reduce the tubular max (*T*_*max*_) for glucose reabsorption which, together with reduced filtered load, would theoretically reduce glucose content within proximal tubule cells. However, in non-diabetic and diabetic mice, empagliflozin did not reduce cortical glucose content when compared to vehicle. Whilst the establishment of an inward glucose gradient at the basolateral surface may occur with SGLT2 inhibition^13^, the reduction in plasma glucose levels with empagliflozin as well as reduced cortical expression of *Slc2a2* (encoding GLUT2) with co-therapy, argues against this possibility. Although unlikely to fully account for unchanged cortical glucose content, an explanation may be enhanced SGLT1-mediated glucose reabsorption, as seen previously under SGLT2 blockade^34^. In this study, kidney mRNA levels of three key gluconeogenic enzymes, *Pck1*, *Fbp1*, and *G6pc*, were not enhanced by empagliflozin suggesting there was no compensatory increase in renal gluconeogenesis. This is in agreement with another study where kidney *Pck1* levels were reduced in Akita/+ mice treated with empagliflozin^21^. Endogenous glucose production (EGP) is enhanced by SGLT2 inhibition in humans^35^,^36^ and, given the abovementioned findings in mice, this may primarily be of hepatic origin. We observed that the diabetes-induced increase in renal gluconeogenic gene expression (*Pck1* and *Fbp1*) was exacerbated by metformin. This may be explained by compensatory EGP from renal sources, if metformin specifically suppresses hepatic gluconeogenesis^37^,^38^, although this remains to be tested in future studies.

SGLT2 inhibition increased both plasma and intrarenal renin activity in diabetic and non-diabetic mice. In line with this, eight-week treatment with empagliflozin in individuals with type 1 diabetes increased circulating angiotensin II levels^9^, albeit, in our study, intrarenal angiotensin II content remained unchanged. Increased RAS activity with SGLT2 inhibition is explained by the expected volume depletion with this class of therapy^39^. Despite a tendency for reduced GFR with SGLT2 inhibition, likely due to increased afferent tone via tubuloglomerular feedback^40^, we cannot rule out relevant increases in efferent tone via activation of the RAS; which could increase intraglomerular pressure. RAS inhibition may also restore glomerular function independent of increased capillary pressure^41^ and thus the benefits afforded by dual RAS-SGLT2 inhibition warrant further study. Indeed, in Dahl salt-sensitive diabetic rats, maximal renoprotection from glomerular injury, renal fibrosis, and proteinuria was achieved when luseogliflozin was combined with the ACE inhibitor, lisinopril^42^.

In our studies, SGLT2 inhibition increased fasting and glucose-stimulated plasma insulin levels, which was most profound in mice that received dual therapy with metformin, and may account for their weight gain over time. This is contrary to the weight loss reported in obese individuals treated with an SGLT2 inhibitor^7^, however, Lin *et al.* demonstrated similar increases in plasma insulin and body weight in *db/db* mice, along with improvements in albuminuria and glomerulosclerosis^24^. Thus, increased body weight cannot account for the only modest renoprotective effects of SGLT2 inhibition seen in our study. Of note, as per empagliflozin, the anti-hyperglycemic agent metformin was unable to prevent albuminuria but attenuated kidney *Fn1* and *Tgfβ* expression. Interestingly, treatment with metformin exacerbated diabetic kidney growth which warrants additional study.

We demonstrated efficacy in our studies by the presence of glucosuria in non-diabetic mice that were treated with empagliflozin. As seen previously in STZ-diabetic, Akita/+ and *db/db* mice^20^,^21^,^24^,^25^, SGLT2 inhibition did not exacerbate diabetes-induced glucosuria, which is explained by the reduction in filtered glucose load equalling the degree of SGLT2 inhibition. Also, given that GFR remained sufficient, empagliflozin was able to reach the brush border membrane of the proximal tubule and exert its intended effects^43^. The difference in blood glucose lowering between empagliflozin mono- and co-treated groups was modest, suggesting that other factors beyond glucose lowering may contribute to the superior renal outcomes seen with dual therapy in this study. We observed that fasting and glucose-stimulated insulin secretion was considerably increased in the co-treated mice, along with increased pancreatic insulin content. Further, the leftward shift in the relationship between plasma glucose levels and urinary glucose excretion with empagliflozin was absent in the co-treated mice. The mechanism(s) underlying these findings and their relationship, if any, to kidney fibrosis requires additional study.

SGLT2 inhibitors are a new class of anti-diabetic agent and human studies in type 1 and 2 diabetes have demonstrated efficacy in blood glucose lowering and acute hemodynamic changes in kidney function^9^,^44^. Long-term clinical studies on the incidence of microvascular complications, such as diabetic nephropathy, are ongoing and will determine whether SGLT2 inhibitors exert benefits that are superior to traditional agents^13^. While we were unable to show considerable improvements in renal function, the expression of some profibrotic genes was reduced with empagliflozin, in line with the effects of first line anti-diabetic agent, metformin. Additional molecular and histological benefits where offered when empagliflozin and metformin were co-administered. Persistent hyperglycemia and albuminuria-onset prior to commencement of treatment in this model rendered our study an interventional rather than a preventative approach. We suggest that a threshold of blood glucose lowering may be required to achieve renoprotection in diabetes, which may differ for individual parameters, as evidenced by some, but not all, features of kidney disease improving with SGLT2 inhibition in our study. Thus, taken together with previous work, we suggest that early, sufficient, and stable blood glucose lowering, possibly with multiple agents, including higher- and/or multiple daily-dosing of SGLT2 inhibition in combination with RAS blockade, may be required to achieve maximal renoprotection in diabetes.

## Methods

### Animals

All procedures were performed in accordance with guidelines from the University of Queensland and the National Health and Medical Research Council of Australia. All experimental protocols on mice were approved by the University of Queensland Animal Ethics Committee. Male BKS.Cg-*Dock7*^*m*^+/+ *Lepr*^*db*^/J (*db/db*) mice and lean, heterozygote controls (*db/m*) were purchased from Jackson laboratories (stock number 000642; Bar Harbor, ME, USA). Mice were housed in an environmentally controlled room (constant temperature 22 °C), with a 12:12 h light-dark cycle and access to standard chow and tap water *ad libitum*. At 10 weeks of age, *db/db* and *db/m* mice were randomized to receive empagliflozin (10 mg/kg/day; provided by Boehringer-Ingelheim, Germany) or vehicle (0.5% hydroxyethylcellulose, Sigma-Aldrich, St. Louis, MO, USA) by oral gavage for 10 weeks, between the hours of 14:00 and 16:00. Additional *db/db* mice were administered the anti-hyper glycemic agent, metformin (250 mg/kg/day; Sigma-Aldrich), or empagliflozin + metformin co-therapy (as per mono-therapy dosages). Body weight and fasting blood glucose were monitored throughout the study. Approximately 24 h after the last treatment (20 weeks of age), mice were fasted for ~4 h and anesthetized with sodium pentobarbital (150 mg/kg ip; Virbac, Milperra, NSW, Australia). Kidneys and pancreata were excised, snap-frozen in liquid nitrogen, or fixed in 10% neutral buffered formalin.

### Food and water intake, and blood and urine collection

At two and six weeks of the treatment period, mice were weighed and placed individually into metabolic cages for 24 h measurements of food and water intake, and urine collection^45^,^46^. Blood samples were collected via tail tipping immediately upon removal from metabolic cages. Mice were acclimatized to the metabolic cages by placing them in for short daylight periods on two separate occasions prior to the 24 h collection.

### Glomerular filtration rate

At week eight, GFR was estimated in conscious mice using the transcutaneous decay of retro-orbitally injected FITC-sinistrin (10 mg/100 g body weight dissolved in 0.9% NaCl), as previously described^47^. Background signal was recorded for one minute, mice were injected under brief inhaled isoflurane anesthesia, and the signal was recorded for 60 min. GFR was calculated using the half-life derived from the rate constant (α_2_) of the single exponential, excretion phase of the curve and a semi-empirical factor. Plasma cystatin C was also measured at the study end, after 10 weeks of treatment (ELISA, BioVendor, Brno, Czech Republic).

### Oral glucose tolerance test

At week eight, an oral glucose tolerance test (OGTT) was performed following a 6 h fast between 08:00–14:00 h^48^. Blood samples were taken via tail tipping prior to (0 min) and following an oral glucose bolus (2 g/kg body wt of 50% w/v D-glucose solution) at 5, 15, 30, 60, and 120 min for determination of plasma glucose and insulin concentrations. The efficacy of insulin secretion was calculated (insulinogenic index; Insulin_30_-Insulin_0_/Glucose_30_-Glucose_0_)^49^. The ratio of fasting plasma glucose-to-insulin ratio and homestatic model of assessment for insulin resistance (HOMA-IR; fasting plasma insulin (μU/ml) × fasting glucose (mmol/L)/22.5) were also calculated.

### Biochemical analyses

Plasma, urinary, and kidney cortical cytosolic (see below for ultracentrifugation; ‘*Western blotting’*) glucose concentrations were measured by the glucose oxidase method (Cayman Chemical, Ann Arbor, MI, USA). Glycated hemoglobin was measured in whole blood samples using an enzymatic assay kit (Crystal Chem Inc., Downers Grove, IL, USA). ELISA kits were used for the measurement of plasma insulin (Crystal Chem Inc.), urinary albumin (Bethyl laboratories, Montgomery, AL, USA), kidney injury molecule-1 (KIM-1; USCN Life Science Inc., Hubei, China), and neutrophil gelatinase-associated lipocalin (NGAL; R&D Systems, Minneapolis, MN, USA). Tissue levels of renin and AngII, and plasma levels of renin were determined via radioimmunoassay (Prosearch International, Malvern, VIC, Australia)^50^.

### Histology

Paraffin embedded kidney and pancreas sections were used for the blinded assessment of renal injury and islet insulin content, respectively. Glomerulosclerotic index (GSI) was evaluated by a semi-quantitative method in 20 glomeruli (×400) per animal using 2 μm kidney sections stained with Periodic acid-Schiff (PAS)^46^ Anti-human collagen IV (1:100 dilution; Abcam, Milton, Cambridge, UK) and anti-human fibronectin (1:500 dilution; Sigma-Aldrich) were used to assess mesangial expansion in 20 glomeruli (×400) and tubulointerstitial fibrosis in ten cortical/outer medullary fields (×100) per animal using 4 μm kidney sections. Total collagen accumulation was assessed in ten cortical/outer medullary fields (×100) per animal using 2 μm sections stained with Masson’s trichrome and 4 μm sections stained with Sirius Red^26^,^46^. Pancreatic insulin content was determined using anti-human insulin (1:100 dilution; R&D Systems Inc., Minneapolis, MN, USA) in at least 32 islets from four to six different mice per group. Immuno-positive staining was revealed with 3,3′-Diaminobenzidine tetrahydrochloride (DAB) solution. All sections were visualized using a Nikon Brightfield or Olympus Virtual Slide microscope. Percent of positive staining for Masson’s trichrome, Sirius Red, or DAB (immunohistochemistry) was determined using NIS-Elements imaging software (Nikon Instruments Inc.) or ImageJ (Fiji distribution package).

### Western blotting

Kidney cortices were homogenized in sucrose (250 mM), triethanolamine (10 mM), and complete protease inhibitor cocktail using a dounce homogenizer. Homogenates were centrifuged at 6,000 × g for 10 min and the supernatant ultracentrifuged at 150,000 × g for 1 h to pellet total cell membrane. Lysates (60 μg for SGLT2 and 10 μg for β-actin) were denatured using 4 × Laemmli buffer (Bio-rad) without β-mercaptoethanol at 65 °C for 15 min for SGLT2 or with β-mercaptoethanol at 95 °C for 5 min for β-actin. Protein was resolved using SDS-PAGE, transferred to PVDF membranes, blocked in 5% skim milk for 1 h, and immunoblotted overnight at 4 °C for SGLT2 (anti-rat, 1:1,000, in-house antibody^51^) or 4 h at room temperature for β-actin (anti-human, 1:5,000, Abcam).

### Real-time qPCR

Total RNA was extracted from kidney cortex using Trizol. cDNA was synthesized using MultiScribe first strand cDNA synthesis kit and real-time qPCR performed using pre-designed Taqman Gene Expression Assays for *Slc5a1* (Mm00451203_m1)*, Slc5a2* (Mm00453831_m1)*, Slc2a2* (Mm00446229_m1)*, Pck1* (Mm01247058_m1), *G6pc* (Mm00839363_m1), *Fbp1* (Mm00490181_m1), *ColIVα1* (Mm01210125_m1)*, Fn1* (Mm01256744_m1)*, Tgfβ1* (Mm01178820_m1)*, Ctgf* (Mm01192932_g1), and *Cd14* (Mm00438094_g1, Life Technologies, Mulgrave, VIC, Australia). Relative gene expression was quantified using the comparative threshold cycle (ΔΔCt) with 18S rRNA (Life Technologies) as the endogenous multiplexed control.

### Statistical analyses

Data were analyzed by one-way ANOVA with Tukey’s post hoc (parametric data) or Kruskal-Wallis one-way ANOVA with Dunn’s post hoc (non-parametric data). Student’s unpaired *t*-test was also performed for comparisons between two groups. Student’s paired *t*-test within each group was performed for blood glucose change from baseline, and body weight change between weeks five and 10 of treatment. Data are expressed as means ± SEM, and *P* < 0.05 was considered significantly different and *P* = 0.05–0.099 was considered a trend.

## Additional Information

**Accession codes:** *Slc5a1* (Mm00451203_m1)*, Slc5a2* (Mm00453831_m1)*, Slc2a2* (Mm00446229_m1)*, Pck1* (Mm01247058_m1), *G6pc* (Mm00839363_m1), *Fbp1* (Mm00490181_m1), *ColIVα1* (Mm01210125_m1)*, Fn1* (Mm01256744_m1)*, Tgfβ1* (Mm01178820_m1)*, Ctgf* (Mm01192932_g1), and *Cd14* (Mm00438094_g1).

**How to cite this article**: Gallo, L. A. *et al.* Once daily administration of the SGLT2 inhibitor, empagliflozin, attenuates markers of renal fibrosis without improving albuminuria in diabetic *db/db* mice. *Sci. Rep.* **6**, 26428; doi: 10.1038/srep26428 (2016).

## Supplementary Material

Supplementary Information

## Figures and Tables

**Table 1:** Body and kidney weight, food and water consumption, and urine output.

	Treatment duration	*db/m* vehicle	*db/m* empa	*db/db* vehicle	*db/db* empa	*db/db* met	*db/db* empa + met
Body weight (g)	Baseline	27.0 ± 0.6	27.3 ± 0.4	41.9 ± 0.9*	42.1 ± 1.0*	40.8 ± 0.9*	41.0 ± 1.0*
	5 weeks	27.8 ± 0.6	27.5 ± 0.4	42.3 ± 1.2*	46.1 ± 1.2*	43.7 ± 1.2*	47.6 ± 1.0*^,†^
	10 weeks	28.8 ± 0.7	28.5 ± 0.3	39.3 ± 1.3^*^	47.4 ± 1.9^*^^,†,δ^	39.3 ± 2.2^*^	50.7 ± 1.3^*^^,†,δ^
Kidney weight (g/mm tibia length × 10^−2^)	10 weeks	1.48 ± 0.06	1.50 ± 0.03	1.87 ± 0.06^*^	1.77 ± 0.05^*^^,δ^	2.12 ± 0.09^*^^,ε^	1.84 ± 0.05^*^^,δ^
Food consumption (g/24 h)	2 weeks	4.95 ± 0.47	5.27 ± 0.32	9.29 ± 1.22^*^	9.10 ± 0.99^*^	8.54 ± 0.55^*^	9.51 ± 0.97^*^
	6 weeks	5.30 ± 0.34	6.27 ± 0.55	9.25 ± 1.00^*^	9.91 ± 1.26^*^	10.05 ± 1.09^*^	9.13 ± 0.78^*^
Water consumption (ml/24 h)	2 weeks	4.24 ± 0.66	4.80 ± 0.49	19.78 ± 2.00^*^	21.74 ± 2.76^*^	17.18 ± 1.55^*^	16.43 ± 0.95^*^
	6 weeks	4.10 ± 0.28	5.62 ± 0.80^∧^	14.42 ± 1.82^*^	14.65 ± 2.51^*^	19.10 ± 2.35^*^	15.25 ± 1.43^*^
Urine production (ml/24 h)	2 weeks	0.26 ± 0.08	0.65 ± 0.15^∧^	13.18 ± 1.78^*^	14.07 ± 2.59^*^	11.40 ± 1.84^*^	10.84 ± 1.70^*^
	6 weeks	0.36 ± 0.08	0.82 ± 0.15^#^	12.89 ± 1.99^*^	13.48 ± 3.16^*^	13.19 ± 1.92^*^	9.80 ± 1.61^*^

Data are means ± SEM (*n* = 6–11/group). ^*^P < 0.05 *vs db/m* vehicle, ^†^*P* < 0.05 *vs db/db* vehicle, ^δ^*P* < 0.05 *vs db/db* metformin within each time point by one-way ANOVA and Tukey’s post hoc. ^#^*P* < 0.05 *vs db/m* vehicle, ^∧^*P* = 0.05–0.07 (trend) *vs db/m* vehicle, ^ε^*P* = 0.05–0.07 (trend) *vs db/db* vehicle within that time point by Student’s unpaired *t*-test. *N.B.* Body weight between treatment weeks 5 and 10, within each group, were compared by Student’s paired *t*-test and described in the Results section.
