# Erratum: Once daily administration of the SGLT2 inhibitor, empagliflozin, attenuates markers of renal fibrosis without improving albuminuria in diabetic *db/db* mice

**DOI:** 10.1038/srep28124

**Published:** 2016-07-07

**Authors:** Linda A. Gallo, Micheal S. Ward, Amelia K. Fotheringham, Aowen Zhuang, Danielle J. Borg, Nicole B. Flemming, Ben M. Harvie, Toni L. Kinneally, Shang-Ming Yeh, Domenica A. McCarthy, Hermann Koepsell, Volker Vallon, Carol Pollock, Usha Panchapakesan, Josephine M. Forbes

Scientific Reports 6: Article number: 2642810.1038/srep26428; published online: 05262016; updated: 07072016

This Article contains errors in the Acknowledgements section.

“The authors acknowledge funding support from the National Health and Medical Council of Australia (1004926).”

should read:

“The authors acknowledge funding support from the National Health and Medical Research Council of Australia (1004926) and Diabetes Australia.”

