# Supplementary material for: Once daily administration of the SGLT2 inhibitor, empagliflozin, attenuates markers of renal fibrosis without improving albuminuria in diabetic db/db mice
Source: Sci Rep. 2016 May 26;6:26428. doi: 10.1038/srep26428 (PMC4881020; doi:10.1038/srep26428)
Supplement: Supplementary Information [file srep26428-s1.doc]

**Supplementary File**

**Once daily administration of the SGLT2 inhibitor, empagliflozin, attenuates markers of renal fibrosis without improving albuminuria in diabetic *db/db* mice.**

Linda A Gallo^1*^, Micheal S Ward^1^, Amelia K Fotheringham^1^, Aowen Zhuang^1^, Danielle J Borg ^1^, Nicole B Flemming^1^, Ben M Harvie^2^, Toni L Kinneally ^3^, Shang-Ming Yeh^4^, Domenica A McCarthy^1^, Hermann Koepsell^5^, Volker Vallon^6,7^, Carol Pollock^8^, Usha Panchapakesan^8^, Josephine M Forbes^1,9^

^1^Glycation and Diabetes, Translational Research Institute, Mater Research Institute-University of Queensland, Woolloongabba, Queensland, Australia; ^2^University of Queensland Biological Resources and ^3^School of Medicine, University of Queensland, St Lucia, Queensland, Australia; ^4^Science and Engineering Faculty, Queensland University of Technology, Brisbane, Queensland, Australia; ^5^Department of Molecular Plant Physiology and Biophysics, Julius-von-Sachs-Institute, University of Würzburg, Würzburg, Bavaria, Germany; ^6^Departments of Medicine and Pharmacology, University of California San Diego, La Jolla, California, USA 92093; ^7^VA San Diego Healthcare System, San Diego, California, USA 92161; ^8^Department of Medicine, Kolling Institute of Medical Research, University of Sydney, St Leonards, New South Wales, Australia; ^9^Mater Clinical School of Medicine, University of Queensland, South Brisbane, Queensland, Australia

***Corresponding author**:

Linda A Gallo

Mater Research Institute-University of Queensland, Translational Research Institute

37 Kent Street Woolloongabba

Australia 4102

Phone: + 61 7 3443 7676

Fax: + 61 7 3443 7779

Email: linda.gallo@mater.uq.edu.au

**Supplementary Figure 1: HOMA-IR and pancreatic insulin content.** (**a**) HOMA-IR; fasting plasma insulin (µU/ml) × fasting glucose (mmol/L) / 22.5 and (**b**) insulin positivity within pancreatic islets in *db/m* (open) and *db/db* (grey) mice. Data are (**a**) means ± SEM (*n*=6-11/group) and (**b**) median ± IQR with min and max values (*n*≥32 islets/group) for insulin positivity. * *P*<0.05 *vs db/m* vehicle, † *P*<0.05 *vs db/db* vehicle, δ *P*<0.05 *vs db/db* metformin, Ψ *P*<0.05 *vs* all other *db/db* groups by (**a**) one-way ANOVA and Tukey’s post hoc or (**b**) Kruskal-Wallis one-way ANOVA and Dunn’s post hoc. Comparisons by Student’s unpaired *t*-test: significance denoted by solid lines.

**Supplementary Figure 2: Relationship between plasma glucose and urinary glucose excretion.** Comparisons between vehicle- and (**a**) empagliflozin-, (**b**) metformin-, or (**c**) empagliflozin + metformin-treated *db/db* mice. Circles (o) vehicle-treated; squares () empagliflozin-treated; triangles (Δ) metformin treated; and diamonds (◊) empagliflozin + metformin co-treated. Data are individual mice with linear regression (*n*=9-10/group).


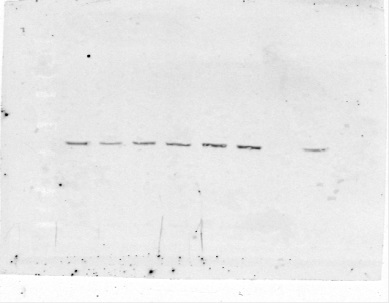

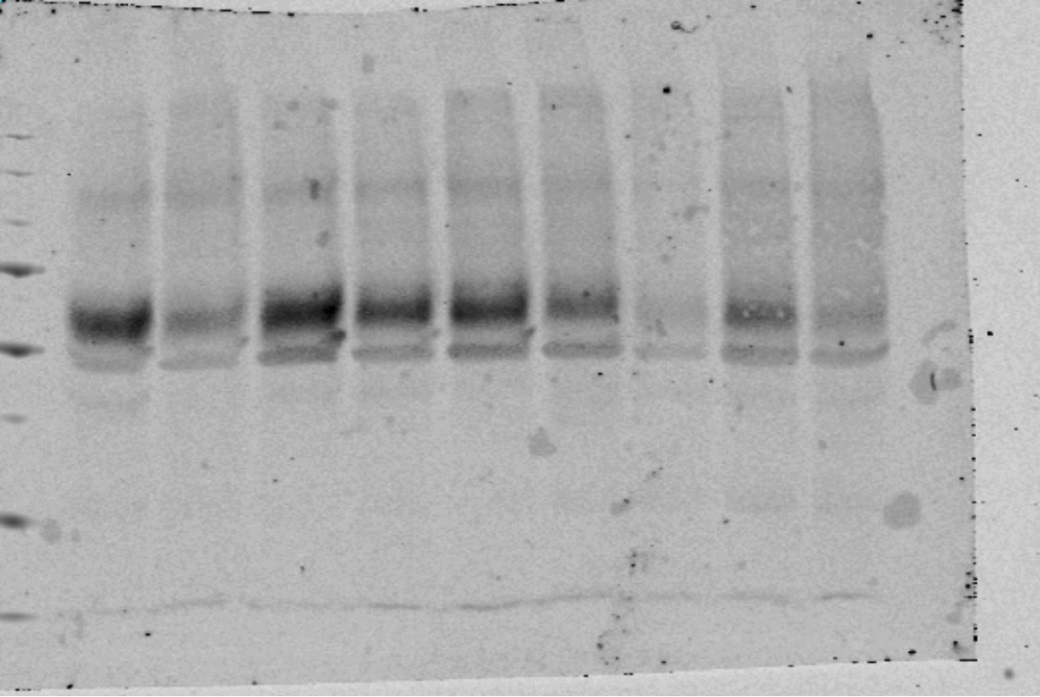


**a**

**b**

**Supplementary Figure 3: Full length of Western immunoblot representatives.** Membrane probed with (**a**) anti-rat SGLT2 and (**b**) anti-human β-actin (*n*=6/group).
